# Capecitabine and temozolomide or temozolomide alone in patients with atypical carcinoids

**DOI:** 10.1007/s12020-025-04171-5

**Published:** 2025-01-24

**Authors:** Linda Galvani, Arianna Zappi, Sara Pusceddu, Fabio Gelsomino, Anna La Salvia, Simone Oldani, Francesco Panzuto, Elisa Andrini, Giuseppe Lamberti, Davide Campana

**Affiliations:** 1 Department of Medical and Surgical Sciences (DIMEC), Alma Mater Studiorum – Università di Bologna, Bologna, Italy; 2 Medical Oncology and Hematology Department, Fondazione IRCCS Istituto Nazionale Tumori, Milan, Italy; 3 Division of Oncology, Department of Oncology and Hematology, University Hospital of Modena, Modena, Italy; 4 Medical Oncology 2, IRCCS Regina Elena National Cancer Institute, Rome, Italy; 5 National Center for Drug Research and Evaluation, National Institute of Health (ISS), Rome, Italy; 6 Department of Medical-Surgical Sciences and Translational Medicine, Sapienza University of Rome, Rome, Italy; 7 Digestive Disease Unit, Sant’Andrea University Hospital, ENETS Center of Excellence, Rome, Italy; 8 Medical Oncology Department, IRCCS Azienda Ospedaliero-Universitaria Sant’Orsola-Malpighi di Bologna, Bologna, Italy

**Keywords:** Lung neuroendocrine neoplasms, Atypical carcinoids, Chemotherapy, TEM, CAPTEM

## Abstract

**Background:**

Lung neuroendocrine neoplasms (NENs) represent about 20% of all lung cancers. Few therapeutic options are available for atypical carcinoids (ACs). Single-agent temozolomide (TEM) is active in lung NENs, but whether the addition of capecitabine (CAPTEM) is associated with improved outcomes, is unknown. We sought to investigate the TEM-based therapies (TEM or CAPTEM) in patients with advanced AC.

**Material and methods:**

This was a retrospective analysis of prospectively collected data from patients with AC of the lung referred to our institution from January 2003 to January 2023 who have received chemotherapy with either TEM or CAPTEM as any line treatment. Primary endpoint was progression free survival (PFS), secondary endpoints included overall response rate (ORR) and overall survival (OS).

**Results:**

In this study we included 31 patients with advanced AC. Median Ki-67 was 14.4% (3–30). CAPTEM in 17 patients (54.8%), while TEM was administered in 14 patients (45.2%). Overall, ORR was 39% (*N* = 12/31, all partial responses), while median PFS and OS were 57.4 months (95%CI: 43.2–71.7) and 24.4 months (95% confidence interval [95%CI]: 14.7–34.1). Median PFS was 33.9 months (15.6–52.1) in the CAPTEM group, while it was 15.5 (7.3–23.8) in the TEM group (*p* = 0.047). When adjusting for potential confounding factors, treatment with TEM vs CAPTEM retained its independent association with an increased risk of progression (HR: 4.01 [95%CI: 1.18–13.68]; *p* = 0.027).

**Conclusions:**

Treatment with CAPTEM is associated with longer PFS than TEM alone in patients with AC. Prospective studies with larger sample size are needed to validate this finding.

## Introduction

Lung neuroendocrine neoplasms (NENs) represent about 20% of all lung cancers and are divided into four subtypes: typical carcinoids (TCs), atypical carcinoids (ACs), large-cell neuroendocrine carcinomas (LCNECs) and small cell lung carcinomas (SCLCs) [1, 2]. Lung NEN account for ~25% of neuroendocrine tumors, and are profoundly different in terms of biology, aggressiveness, and prognosis [3, 4]. TCs are defined as low-grade NENs, with a mitotic count of 0–2 per 10 high power fields (HPF), no histologic evidence of necrosis and are generally characterized by a low growth rate. ACs are considered intermediate-grade lung NENs, defined by a mitotic count ranging from 2 to 10 mitosis per 10 HPF and by the presence of focal necrosis. ACs are less frequent than TCs but more aggressive. LCNECs and SCLCs are high-grade lung NENs, characterized by a strongly aggressive behavior and a dismal prognosis. In particular, SCLC accounts for ~13–15% of all lung NENs and is the most aggressive among them, given its very high mitotic rate and Ki67 proliferation index (usually higher than 70%) [1, 2]. Very few options are available for advanced lung NENs, especially for TCs and ACs [5–7]. Indeed, the selective mTOR inhibitor everolimus is the only approved treatment for TCs and ACs [8–10]. In addition, somatostatin analogs are often administered in somatostatin receptor-positive TCs and ACs patients, despite randomized trials are lacking [11, 12]. In this scenario, treatment options for patients with TCs and ACs are urgently needed. The association of capecitabine and temozolomide (CAPTEM) is routinely used for the treatment of pancreatic NENs thanks to observed objective response rates ranging from 33 to 70%, median progression-free survival ranging from 18 months to 22.7 months [13–15], and the superiority of this combination if compared to temozolomide (TEM) monotherapy in terms of overall survival (OS) and progression free survival (PFS) [16]. Nonetheless, superiority of the addition of CAPTEM to TEM alone has not been explored in advanced lung NENs. We thus sought to assess the efficacy of CAPTEM compared to TEM alone in patients with advanced ACs.

## Materials and methods

### Patient selection

The study was a retrospective analysis of a prospective database of patients affected by AC referred to our center from January 2003 to January 2023 and which have received chemotherapy with either TEM or CAPTEM as any line of treatment. The study was approved by local IRB (Comitato Etico indipendente, IRCCS Policlinico Sant’Orsola-Malpighi of Bologna, protocol code: SOCRATE, 787/2019/Oss/AOUBo) and was conducted in accordance with the principles of the Declaration of Helsinki (revision of Edinburgh, 2000). ACs were defined according to WHO 2015 classification after revision by a NEN-dedicated pathologist. Patients with a diagnosis of LCNEC, SCLC and TC, and those in which the diagnosis of AC was not confirmed after pathology revision were excluded.

### Methods

Demographic, medical history, and pathology data were retrospectively collected by patients’ file revision. Date and stage of initial diagnosis, histology grade, Ki67 proliferation index and mitotic count, number of previous treatments, date of start and end for CAPTEM or TEM treatment and of disease progression, the date of last follow-up or death were extracted. Radiological disease assessment performed by either computed tomography (CT) or positron emission tomography (PET) was allowed, according to local clinical practice. The primary objective of the study was to compare the progression-free survival of patients receiving therapy with temozolomide (TEM) alone and the combination of temozolomide and capecitabine (CAPTEM). The secondary objectives included activity and efficacy, by evaluating the objective response rate (ORR) and overall survival (OS), respectively. Response to treatment was assessed according to RECIST v1.1 criteria, by local investigator [17].

### Statistical analysis

Categorical variables were presented as absolute numbers and proportions and compared using Fisher’s test or Chi-squared test, as appropriate. Continuous variables were reported as median and range and compared using the Mann-Whitney test. The ORR was defined as the proportion of complete responses (CR) and partial responses (PR) among the total evaluable cases according to RECIST v1.1 criteria. The disease control rate (DCR) was defined as the proportion of CR, PR, and stable disease (SD) among the total evaluable cases according to RECIST v1.1 criteria. PFS was defined as the time from treatment initiation to RECIST-defined disease progression (PD) or death from any cause, whichever occurred first. OS was defined as the time from treatment initiation to death from any cause. Survival times were estimated using the Kaplan–Meier method and reported in months, with a 95% confidence interval (CI) estimated using the Greenwood formula. The log-rank method was used to compare survival curves. Risk factors were analyzed using univariate and multivariate analysis with the Cox proportional-hazards method, and hazard ratios (HR) with 95% CI were reported. Because of the relatively small sample size, only variables without missing values were fitted in the multivariate model, which was performed with the forward stepwise method. The receiver-operating-characteristic (ROC) curve was used to determine the best Ki67 cutoff for predicting response, with the optimal value estimated using Youden’s statistics. Statistical significance was defined as a *p* < 0.05. All statistical analyses were performed using IBM SPSS Statistics v.22.

## Results

### Study population

A total of 31 patients affected by advanced ACs were included, 20 (64.5%) female and 11 male (35.5%). The mean age at diagnosis was 59 years (range: 17–75) while the median age at therapy start was 62.8 years (range: 27–76). Mean Ki67 (available in 29 patients) was 14.4% (range: 3–30%). Before treatment start, disease was limited to the thorax in 5 patients (16.1%), in 9 liver metastases were present (29%), and in 17 there were extrahepatic metastases (54.8%). Baseline PET imaging was performed with 18F-fluoro-2-deoxy-d-glucose (FDG) in 23 patients and in 19 (82.6%) pathological uptake was demonstrated, whereas 29 patients underwent somatostatin receptor imaging (SRI) with either 68Ga-DOTATOC or 68Ga-DOTANOC PET/CT scan which demonstrated pathological uptake in 21 (72.4%) cases. Fourteen patients (45.2%) received TEM 200 mg/m^2^ days 1–5 every 28 days, while 17 patients (54.8%) received CAPTEM as follows: capecitabine 750 mg/m^2^ twice daily days 1–14 and temozolomide 200 mg/m^2^ days 10–14 every 28 days. Six patients (19.3%) received TEM and 7 (22.6%) CAPTEM as first line therapy. Patient characteristics are summarized in Table 1.

**Table 1:** Patient clinical and pathological features

	Study population	CAPTEM n. (%)	TEM n. (%)	*p*
Total	31	17 (55%)	14 (45%)	
Sex				
Male	11 (35.5%)	7 (41.2%)	4 (28.6%)	0.465
Female	20 (64.5%)	10 (58.8%)	10 (71.4%)	
Age at therapy (mean and range)	62 (27–76)	65 (47–74)	60 (27–76)	0.518
Ki67% (mean and range)	14.4 (3–30)	15.6 (5–30)	13.0 (3–30)	0.329
Extension disease				
Toracic	5 (16.1%)	2 (11.8%)	3 (21.4%)	0.147
Liver	9 (29.0%)	3 (17.6%)	6 (42.9%)	
ExtraLiver	17 (54.8%)	12 (70.6%)	5 (35.7%)	
PET-FDG				
Positive	19 (61.3%)	12 (70.6%)	7 (50.0%)	0.022
Negative	4 (12.9%)	0	4 (28.6%)	
Missing	8 (25.8%)	5 (29.4%)	3 (21.4%)	
PET-Ga				
Positive	21 (67.7%)	10 (58.8%)	11 (78.6%)	0.474
Negative	8 (25.8%)	5 (29.4%)	3 (21.4%)	
Missing	2 (6.5%)	2 (11.8%)	0	
First Line	13 (41.9%)	7 (22.6%)	6 (19.3%)	0.606

### Progression-free survival (PFS)

**Fig. 1:**
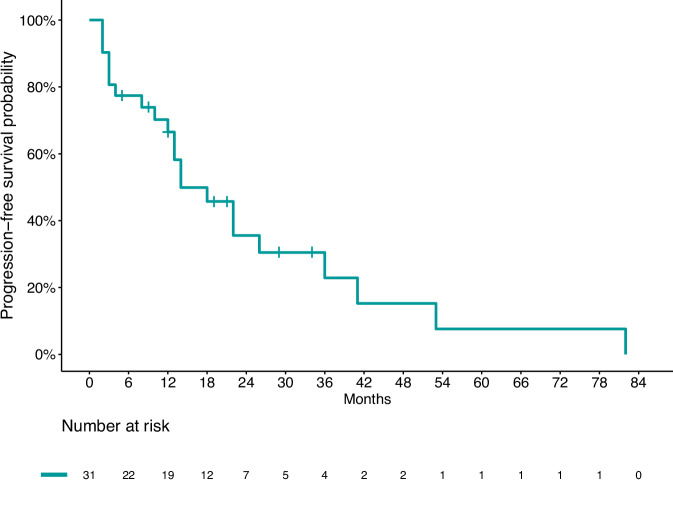
Kaplan–Meier estimates of progression-free survival in the overall population

**Fig. 2:**
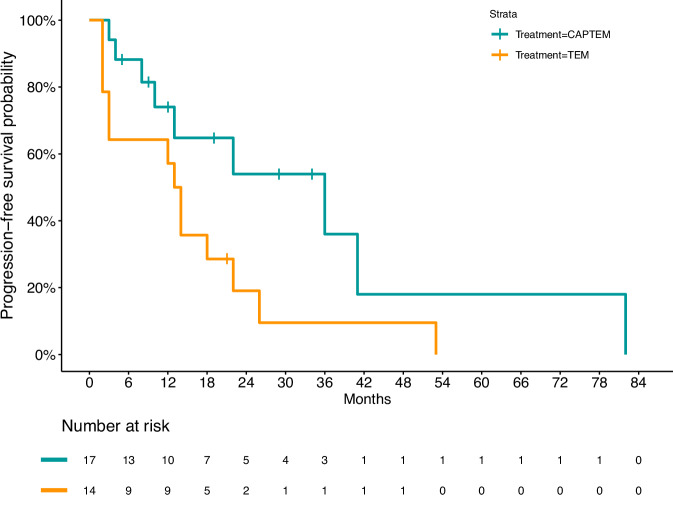
Kaplan–Meier estimates of progression-free survival by treatment. CAPTEM Capecitabine and temozolomide, TEM temozolomide

Overall, median PFS (mPFS) was 24.4 months (95% CI: 14.7–34.1) (Fig. 1). mPFS was longer in patients who received CAPTEM (33.9 months, 95% IC 15.6–52.1) compared to those who received TEM (15.5 months, 95% IC 7.3–23.8; *p* = 0.047) (Fig. 2).

There was no significant difference in mPFS according to sex (male, 28.6 months vs female, 22.5 months; *p* = 0.969), disease extent (thoracic disease, 40.0 months vs liver metastases 12.2 months vs extrahepatic metastases, 27.8; *p* = 0.088), FDG PET uptake (negative: 16.0 months vs positive 21.2 months; *p* = 0.457) or SRI uptake (negative: 17.2 months, positive: 28.2 months; *p* = 0.395) or line of therapy (first line: 19.0 months vs subsequent line: 29.3 months; *p* = 0.427). Risk factors for PFS were reported in Table 2. At univariate analysis, TEM therapy was associated with a trend towards an increased risk for progression, although not reaching statistical significance (HR: 2.35, *p* = 0.06). Nonetheless, after adjusting for potential confounding factors, treatment with TEM vs CAPTEM was independently associated with an increased risk for progression (HR: 4.01; 95%CI: 1.18–13.68; *p* = 0.027).

**Table 2:** Univariate and multivariate analysis of the risk for progression or death by Cox proportional hazards method

	Univariate			Multivariate		
	HR	95%	*p*	HR	95%	*p*
Sex						
Female	1					
Male	0.98	0.39–2.45	0.970			ns
Ki67%	1.03	0.97–1.09	0.288	-	-	-
Extension disease						
Thoracic	1			1		
Liver	4.40	0.87–22.24	0.073	5.06	0.95–27.0	0.057
Extraliver	1.98	0.43–9.10	0.381	5.62	0.94–33.61	0.059
PET-FDG						
Negative	1			-	-	-
Positive	0.65	0.20–2.10	0.474			
PET-Ga						
Negative	1			-	-	-
Positive	0.67	0.26–1.74	0.411			
Treatment line						
Second or later	1					
First	1.42	0.58–3.47	0.440			ns
Treatment						
CAPTEM	1					
TEM	2.35	0.96–5.74	0.06	4.01	1.18–13.68	0.027

Overall, the ORR was 38.7% (12 PRs according to RECIST criteria). PR occurred in 8 patients treated with CAPTEM and 4 patients treated with TEM (47.5% vs 28.6%, respectively; *p* = 0.206).

### Overall survival (OS)

**Fig. 3:**
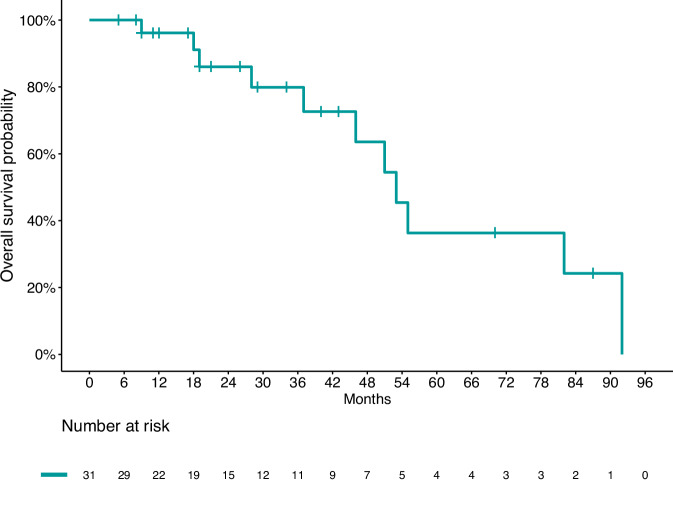
Kaplan–Meier estimates of overall survival in the overall population

**Fig. 4:**
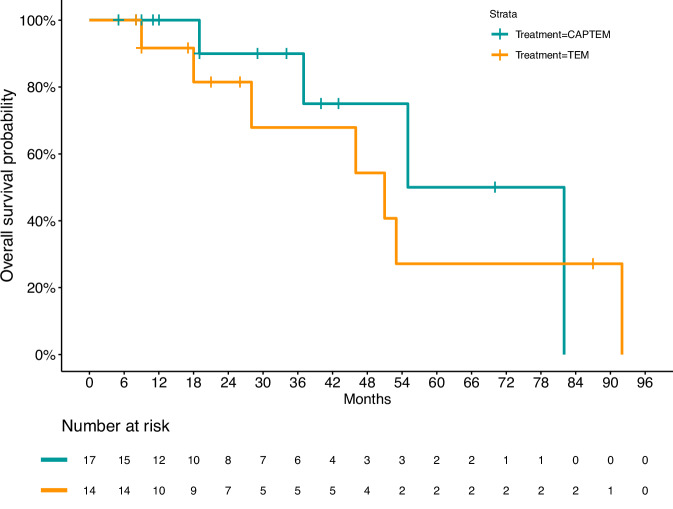
Kaplan–Meier estimates of overall survival by treatment. CAPTEM Capecitabine and temozolomide, TEM temozolomide

Overall median OS in the whole population was 57.4 months (95% IC: 43.2–71.7) (Fig. 3). No difference in OS was observed by sex (*p* = 0.682), disease extent (*p* = 0.641), FDG-PET uptake (*p* = 0.782), SRI positivity (*P* = 0.396), or treatment (*P* = 0.532) (Fig. 4).

## Conclusions

To the best of our knowledge, this retrospective study is the first that compared two distinct chemotherapy approaches based on CAPTEM or TEM alone in a homogenous group of patients with advanced AC. We found that CAPTEM was associated with a doubled mPFS as compared to those who received TEM alone. Overall, an ORR of 47.5% with CAPTEM and of 28.6% with TEM was observed, although non significantly different among the two group.

Currently, the treatment of TCs and ACs is not well-established and primarily involves the use of somatostatin analogs, everolimus, and chemotherapy.

A previous retrospective study evaluated the effectiveness of somatostatin analogs in 67 patients with lung carcinoids, of which 41 were ACs. The study reported a mPFS of 17.4 months (95% CI: 8.7–26.0), and stable disease was the best overall response in 77% of the cases [18].

Recent updates of National Cancer Institute Cancer Network (NCCN) guidelines [19] recommend somatostatin analogs as first-line for advanced somatostatin receptor-positive and/or symptomatic lung carcinoids as an alternative to observation.

The RADIANT-4 study was a phase III randomized controlled trial of everolimus vs placebo and best supportive care (including somatostatin analogs) in patients with advanced non-functional lung and gastrointestinal neuroendocrine tumors [9]. Of the 302 enrolled patients, 90 had lung NENs. The observed mPFS was significantly longer in the everolimus than in the placebo arm (11 months vs 3.9 months).

Chemotherapy is recommended for the treatment of ACs or in TCs/ACs progressing on somatostatin analogs as an alternative to everolimus [1]. TEM, which is among the recommended drugs, has shown modest activity as single agent in a retrospective study which included 31 patients with TC (14 patients) and AC (15 patients) and reported a mPFS of 5.3 months and an ORR of 14% [20]. The only prospective evidence comes from the phase II single-arm ATLANT study, which investigated the association between the somatostatin analog lanreotide autogel and TEM in progressive lung and thymic carcinoid patients [21]. The study was formally negative since the disease control rate at 9 months, primary endpoint of the study, was 35.0%, with a lower bound of the CI lower than the clinically relevant threshold of ≥30%; in this study mPFS was ~9.3 months.

Nonetheless, ACs is a heterogenous group of tumors, which includes more indolent tumors alongside more aggressive ones, referred to as “supracarcinoids”, which could benefit from treatment intensifications with chemotherapy combinations, such as platinum-based chemotherapy or CAPTEM [22]. Platinum-etoposide chemotherapy demonstrated modest efficacy in a respective series of 13 patients with TC or AC, owing it to an ORR of 23% and a mPFS of only 7 months [23]. Other platinum-based (cisplatin or oxaliplatin) schedules were investigated in NEN, but only few patients with TC or AC were included in these studies [24–26].

On the other hand, CAPTEM has been extensively studied in gastroenteropancreatic neuroendocrine neoplasms [14, 27–31] and proved to be superior to TEM alone in the prospective phase II randomized ECOG-ACRIN E2211 trial (PFS, primary endpoint of the study, 22.7 months vs 14.4 months, respectively; HR: 0.58) [14]. However, similarly to what observed in our study, OS was not different between the two treatment arms [14]. In a review with a total of 1912 NEN patients treated with CAPTEM regimen, with only two studies involving exclusively patients with lung NENs, DCR was 77% (range 43.5–100%). The mPFS was reported in 35 of the included studies, ranging from 4 to 38.5 months, while mOS was reported in 32 studies, ranging from 8 to 103 months. In 8 studies mOS was not reached [32]. In the setting of lung carcinoids, a small retrospective study which included 20 patients (14 with TC, 5 with AC) who received CAPTEM, reported a median OS of 68 months, a mPFS of 13 months, and an ORR of 30%, with a disease control rate of 85% [33]. Furthermore, in another small retrospective work, CAPTEM was administered to 33 patients with advanced lung carcinoids (61% of which were ACs), resulting in a mPFS of 9 months and a mOS of 30.4 months [34]. In a homogenous population of 31 patients with AC, we observed an encouraging increased activity of CAPTEM over TEM alone, suggesting that the combination might be considered as an option in these patients.

Indeed, CAPTEM is a well-tolerated treatment [27, 28], with few grade 3–4 toxicities, namely bone marrow toxicities and common mild non-hematological adverse events, such as mucositis, fatigue, nausea, and diarrhea.

An intrinsic limitation of our study is its retrospective nature, which also affected the availability of safety data. Nonetheless, in the setting of rare cancers, retrospective data are of paramount importance to investigate new treatment strategies. Despite the relatively small sample size, this is the largest study of CAPTEM in ACs, to the best of our knowledge. In addition, it must be noted that the absence of significant difference in baseline characteristics between the two treatment groups might be due to a low power of statistics test secondary to the small sample size (e.g., disease extent).

There is an urgent need for new therapeutic options in patients with lung carcinoids, especially ACs. Our findings support the use of CAPTEM in patients with AC, even though larger prospective confirmatory studies are needed to determine the optimal duration of CAPTEM treatment and identify the most effective therapy sequence.

In the present study, the efficacy of CAPTEM seems greater than that of TEM alone in patients with advanced ACs in terms of PFS. Prospective studies with bigger sample size are needed to confirm these results.
